# Low oxygen eddies in the eastern tropical North Atlantic: Implications for N_2_O cycling

**DOI:** 10.1038/s41598-017-04745-y

**Published:** 2017-07-06

**Authors:** D. S. Grundle, C. R. Löscher, G. Krahmann, M. A. Altabet, H. W. Bange, J. Karstensen, A. Körtzinger, B. Fiedler

**Affiliations:** 1 GEOMAR Helmholtz Centre for Ocean Research Kiel, Kiel, Germany; 2 Bermuda Institute of Ocean Sciences, Saint George’s, Bermuda; 3 University of Southern Denmark, Odense, Denmark; 4 School for Marine Science and Technology, University of Massachusetts Dartmouth, Dartmouth, USA

## Abstract

Nitrous oxide (N_2_O) is a climate relevant trace gas, and its production in the ocean generally increases under suboxic conditions. The Atlantic Ocean is well ventilated, and unlike the major oxygen minimum zones (OMZ) of the Pacific and Indian Oceans, dissolved oxygen and N_2_O concentrations in the Atlantic OMZ are relatively high and low, respectively. This study, however, demonstrates that recently discovered low oxygen eddies in the eastern tropical North Atlantic (ETNA) can produce N_2_O concentrations much higher (up to 115 nmol L^−1^) than those previously reported for the Atlantic Ocean, and which are within the range of the highest concentrations found in the open-ocean OMZs of the Pacific and Indian Oceans. N_2_O isotope and isotopomer signatures, as well as molecular genetic results, also point towards a major shift in the N_2_O cycling pathway in the core of the low oxygen eddy discussed here, and we report the first evidence for potential N_2_O cycling via the denitrification pathway in the open Atlantic Ocean. Finally, we consider the implications of low oxygen eddies for bulk, upper water column N_2_O at the regional scale, and point out the possible need for a reevaluation of how we view N_2_O cycling in the ETNA.

## Introduction

Nitrous oxide (N_2_O) is an important climate-relevant trace gas and the oceans are thought to contribute approximately 35% of all natural sources to the atmosphere^1^. In the troposphere N_2_O acts as a greenhouse gas and has a global warming potential which is ~300 times that of CO_2_ over 100 year time-scales^2^. Due to its relative chemical stability, N_2_O also survives transport to the stratosphere where it undergoes photochemical reactions that destroy ozone^3^. In the oceans, N_2_O is produced via the nitrification and denitrification pathways. During nitrification, N_2_O can be produced as a by-product during ammonia oxidation (AO), or through nitrifier-denitrification whereby AO organisms reduce nitrite (NO_2_^−^) to N_2_O. In oxygenated waters, nitrification-N_2_O yields (i.e. those arising from either AO or nitrifier-denitrification) are small, however, under low DO concentrations nitrification-N_2_O yields may increase substantially^4,5^. As DO concentrations approach anoxic conditions, denitrification can also be ‘turned on’, and although it can both produce and consume N_2_O, net denitrification yields up to 2% have been observed^6,7^.

Due to the sensitivity of N_2_O production to low oxygen conditions, the greatest oceanic accumulations, and likely the largest fluxes to the atmosphere, occur in the vicinity of suboxic and anoxic oxygen minimum zones (OMZs), such as those found in the Arabian Sea and the eastern tropical Pacific^8–10^. In comparison, the more ventilated Atlantic Ocean, with higher oxygen concentrations^11,12^, has lower N_2_O production and concentrations^13,14^. Here, we demonstrate for the first time, however, that recently discovered low oxygen mesoscale eddies in the otherwise oxygenated tropical North Atlantic^15^, can induce substantial increases in N_2_O production and cause shifts in the N_2_O cycling pathways.

**Figure 1:**
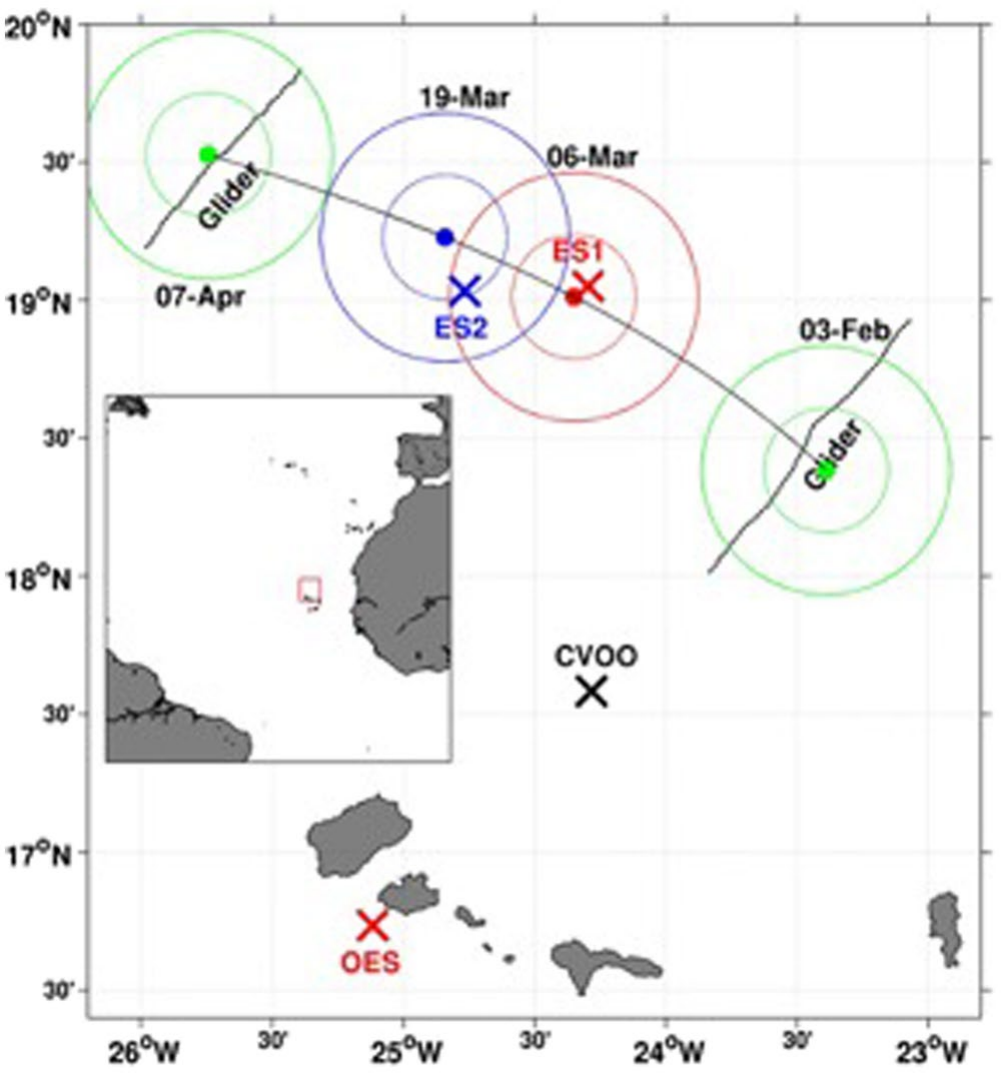
Locations of the relevant sampling sites. The Cape Verde Ocean Observatory (CVOO), outside eddy station (OES), eddy station 1 (ES1) and eddy station 2 (ES2) are marked with crosses, while the IFM12 and IFM13 glider surveys are indicated by dashed lines. The outer solid red and blue circles represent the position and area of the suboxic eddy during the ES1 and ES2 sampling events, respectively, while the outer solid green circles represent the location of the suboxic eddy during glider surveys (see description in *Methods* section). Note: the inner green, red and blue circles represent the area of the low DO core of the eddy. This map was created using Mathworks Matlab version R2014b (http://www.mathworks.com), and the coastline data are from the GSHHS (Global Self-consistent, Hierarchical, High-resolution Shorelines) data set published for free use by NOAA (https://www.ngdc.noaa.gov/mgg/shorlines/gshhs.html).

The OMZ in the North Atlantic Ocean is rather well ventilated, and lowest DO concentrations are around 40 µmol kg^−1 ^^11,12^. Recently, however, coherent mesoscale cyclonic eddies (CE) and anticyclonic mode water eddies (ACME) in the eastern tropical North Atlantic (ETNA), which form off the coast of west Africa along topographical features such as headlands, and then propagate westwards past the Cape Verde Islands^16^, have been shown to create extremely low DO concentrations (as low as ~2 µmol kg^−1^)^15^. The low DO concentrations inside the eddy have the potential to have important implications for biogeochemical processes, including N_2_O cycling. Until recently, however, these potential implications have not been studied, as observations have been opportunistic and most have originated from moored and glider based sensors at the Cape Verde Ocean Observatory (CVOO; Fig. 1). In early 2014, however, a dedicated multi-disciplinary shipboard survey of one of these eddies (hereinafter referred to as ‘suboxic eddy’) was conducted. This survey allowed us to investigate how N_2_O cycling may be impacted by low oxygen eddies in the ETNA (sampling parameters and stations are outlined in the *Methods* section). The results from this work not only demonstrate the potential importance of low oxygen eddies as a source of N_2_O, they also provide insights into how N_2_O cycling in the ETNA may respond to future DO decreases.

## Results and Discussion

### Dissolved oxygen and nitrous oxide concentrations

**Figure 2:**
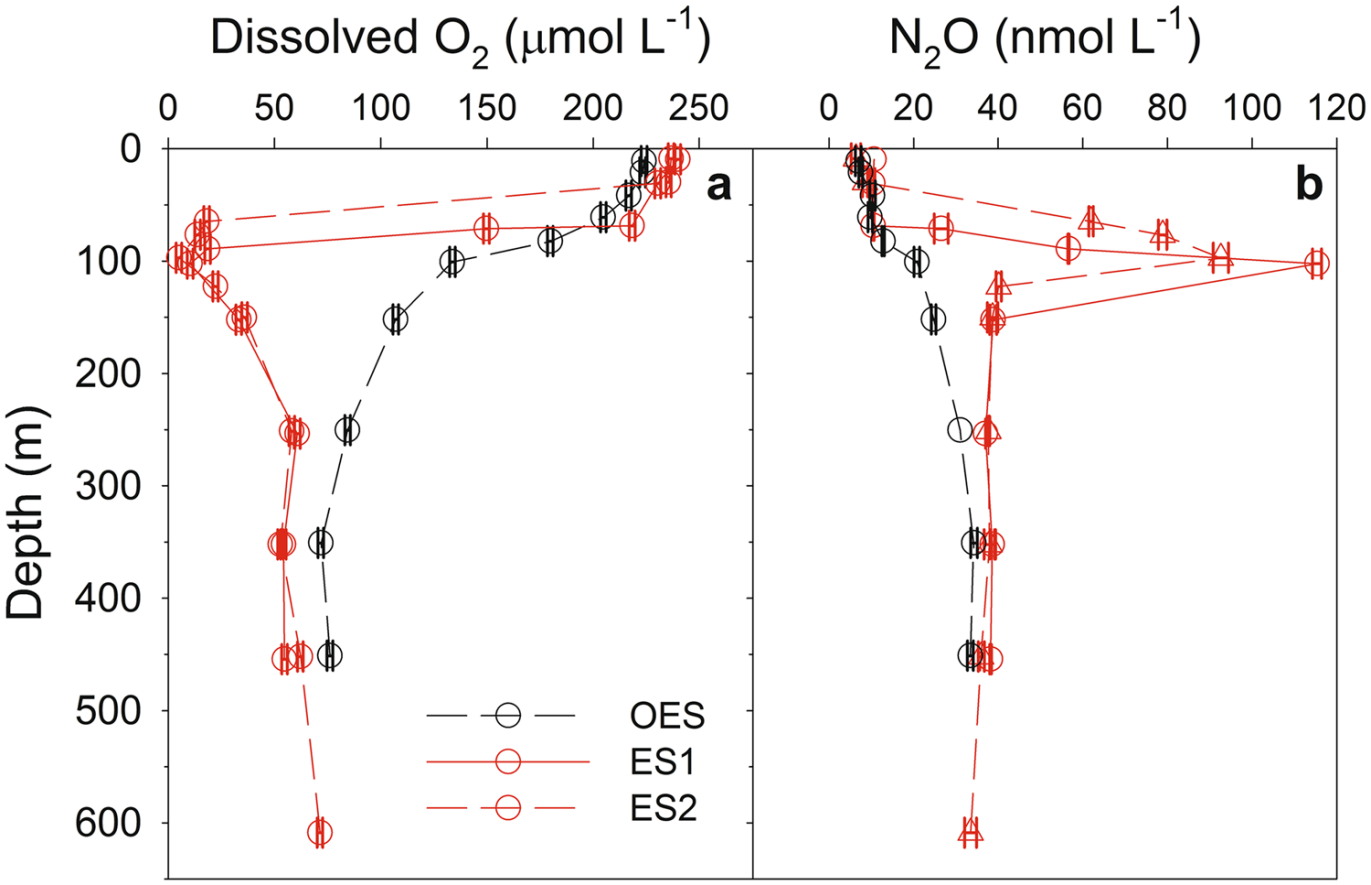
Vertical distributions of dissolved oxygen and N_2_O concentrations at the out-of-eddy station (OES), and at eddy stations 1 and 2 (ES1 and ES2, respectively). (**a**) Discrete depth dissolved oxygen (DO) concentrations measured with the CTD-DO sensor at each of our sampling depths. The error bars represent the average propagation of error associated with our DO measurements (see *Methods* section). (**b**) Discrete depth N_2_O concentrations. The error bars represent the standard deviation of duplicate N_2_O concentration measurements. Due to the loss of duplicated samples, standard deviations are not reported for 10 m depth at ES1, and 250 m depth at OES.

**Figure 3:**
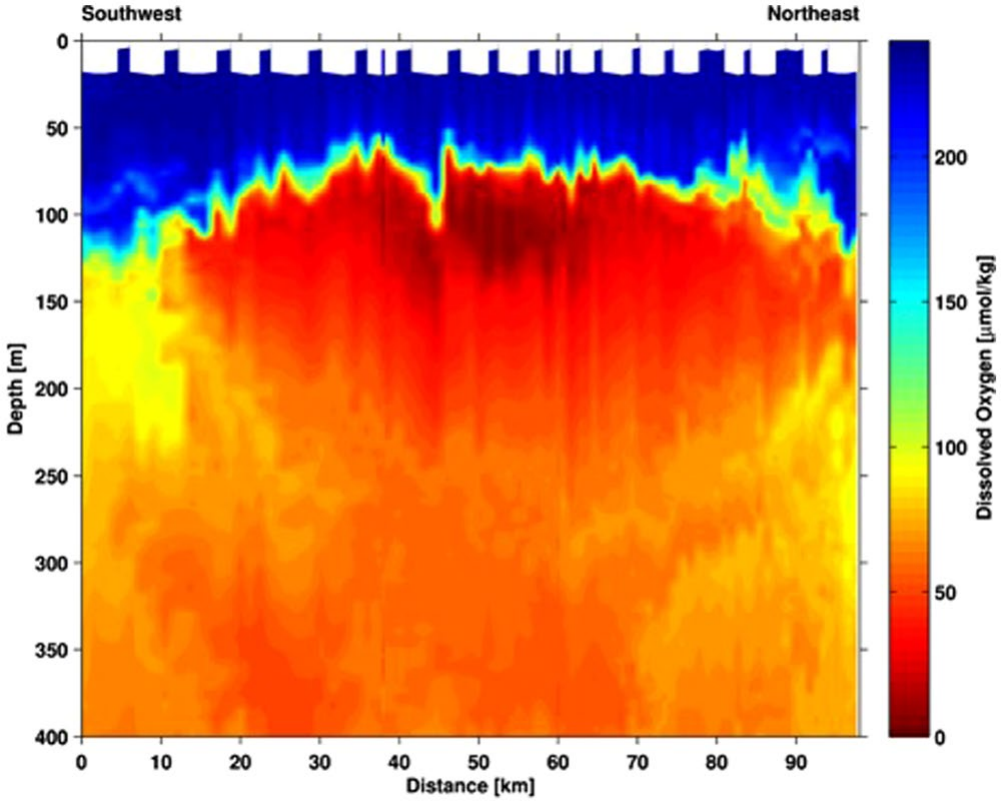
Dissolved oxygen concentrations as measured during the IFM13 glider survey on April 7^th^, 2014.

This study was part of a multi-disciplinary investigation of the suboxic eddy, and here we focus on the implications of the low DO concentrations inside the suboxic eddy for N_2_O processes. The physical characteristics of the eddy and other biological and biogeochemical processes are discussed elsewhere^16–21^. Sampling was conducted at two stations inside the eddy (eddy station 1 and 2; ES1 and ES2) and at an out-of-eddy reference station (OES; Fig. 1). Results show that the suboxic eddy was characterized by DO concentrations that were much lower than in the surrounding waters. For example, the lowest DO concentration we observed at OES was 72 µmol L^−1^ (Fig. 2a), and while this is somewhat higher than the lowest DO concentrations found in the ETNA (~40 µmol kg^−1^), it is within the range of the lowest DO concentrations often found in the region of the CVOO time-series station^22^. In contrast, the lowest DO concentrations at ES1 and ES2 were 10 and 5 µmol L^−1^, respectively, at 100 m (Fig. 2a). Glider surveys of the suboxic eddy also found lows of ~5 µmol O_2_ L^−1^ at 100 m depth (Fig. 3). The suboxic eddy sampled during this study was an ACME, and our observations of a shallow OMZ, with DO concentrations much lower than the ‘typical’ background conditions, are consistent with previous observations of low oxygen ACMEs and CEs which have transited through the CVOO time-series region^15^. The low oxygen conditions inside of these eddies likely result from increased remineralization below the mixed layer, resulting from high primary production and subsequent particulate matter export from the euphotic zone^15,16^. The high primary production is thought to be driven by enhanced upward vertical nutrient fluxes^17^. Indeed, in the suboxic eddy discussed here, mixed layer nutrient concentrations were higher inside vs. outside the eddy^17^, and primary production^21^ and particulate organic carbon fluxes^20^ were estimated to be up to three times higher inside the eddy compared to the surrounding waters.

Similar to the DO results, we observed large perturbations to the N_2_O conditions inside vs. outside of the suboxic eddy. The highest N_2_O concentration at OES (34.2 nmol L^−1^; Fig. 2b) was within the range of the highest concentrations previously reported for the North Atlantic^14,23^, but somewhat lower than the highest concentration found in the eastern tropical South Atlantic (49 nmol L^−1^)^24^. Corresponding to the vertical depth range of low DO, N_2_O concentrations much higher than those previously reported for the North Atlantic were found inside the suboxic eddy, with values as high as 115 nmol L^−1^ within the ES1 OMZ (100 m depth; Fig. 2b). The high N_2_O concentrations we observed in the core of the suboxic eddy are within the range of many of the highest values reported for the eastern tropical Pacific^25–29^ and open Arabian Sea^30,31^, although concentrations as high as up to ~500 nmol N_2_O L^−1^ have been reported for the coastal regions of the eastern tropical South Pacific off of Chile^32^. The observations reported here demonstrate that N_2_O concentrations within ETNA suboxic eddies can reach levels comparable to those from regions that are characterized by well defined OMZs where DO concentrations are permanently low (e.g. eastern tropical Pacific and Arabian Sea), and which are considered to be major sources of oceanic N_2_O. To this end, low oxygen eddies in the ETNA may prove to be an important but previously unrecognized source of N_2_O.

**Figure 4:**
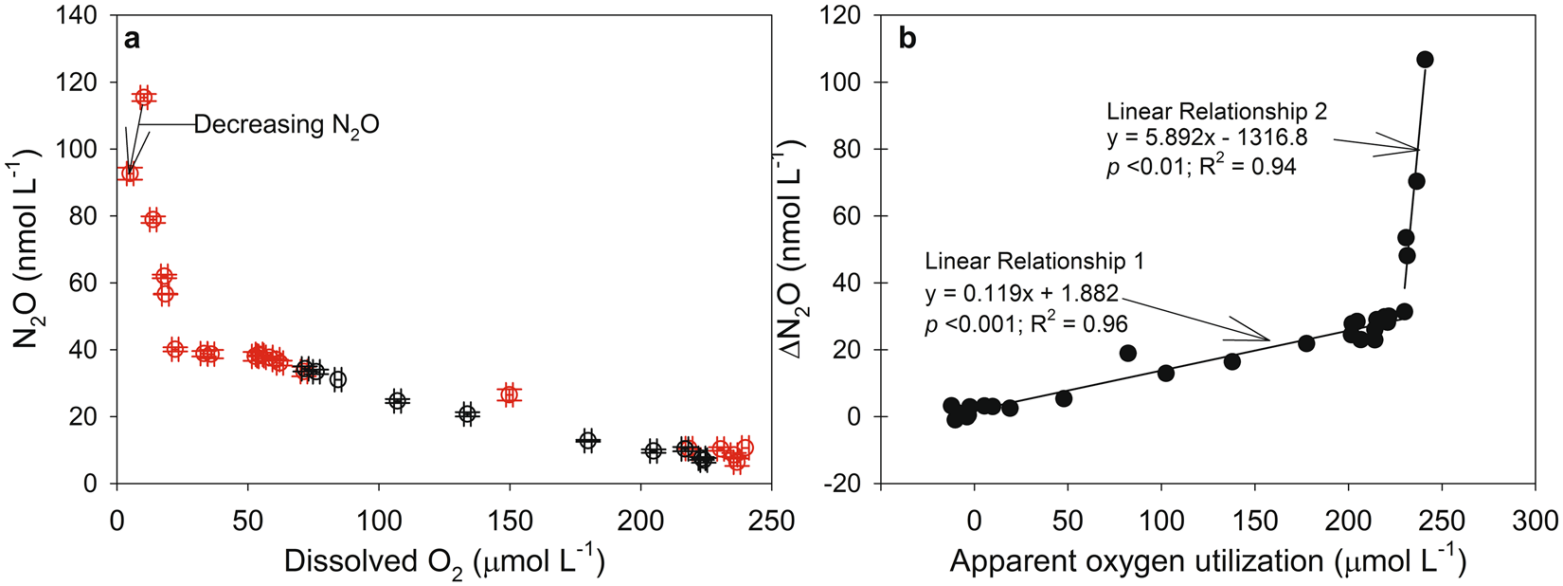
Relationship between nitrous oxide and dissolved oxygen (DO) parameters. (**a**) Nitrous oxide vs. DO concentrations from ES1 and ES2 (red circles) and from OES (black circles). The x-axis error bars represent the average propagation of error associated with our DO measurements (see *Methods* section) and the y-axis error bars represent the standard deviation of duplicate N_2_O concentration measurements. (**b**) ΔN_2_O vs. apparent oxygen utilization (all data pooled; note: data point from the DO minimum at ES2 where the N_2_O concentration decreased is omitted). The solid lines are linear regressions and the results from the linear regression analyses are included.

A plot of all N_2_O and DO concentration data showed these two variables to be inversely correlated down to a DO concentration of 10 µmol L^−1^ (Fig. 4a). Between 10 and 5 µmol O_2_ L^−1^, however, this trend appears that it may have begun to reverse as the N_2_O concentration decreased from 115 to 92.7 nmol L^−1^ (Fig. 4a). It is important to note that the observation of a decrease in N_2_O concentration between 10 and 5 µmol O_2_ L^−1^ was based on sampling conducted almost two weeks apart, and, as such, the decrease may have been due to N_2_O diffusing out of the DO minimum/N_2_O maximum in the period between our two sampling events. If N_2_O was diffusing across a high to low concentration gradient, then DO would have also likely been diffusing from high to low concentrations (i.e. into the DO minimum), and this would have started to erode the extremely low DO concentrations we observed. A glider survey of the eddy on April 7^th^ 2014 (i.e. three weeks after our ES2 sampling date) showed that the low DO eddy core was still stable and intact (Fig. 3). To this end, it seems unlikely that diffusion was a major contributor to the decrease in N_2_O we observed between 10 and 5 µmol O_2_ L^−1^. A shift from net N_2_O production to net N_2_O consumption is another possible explanation for the decrease in N_2_O concentrations between 10 and 5 µmol O_2_ L^−1^. It is important to note, however, that the suggestion of a switch from net N_2_O production to net N_2_O consumption should be treated with caution given that it is based on a single observation of N_2_O decreasing between 10 and 5 µmol O_2_ L^−1^. Still, it is not an unreasonable proposition as previous results have also shown evidence for a transition from net production to net consumption below 10 µmol O_2_ L^−1 ^^33^.

At DO concentrations ≥10 µmol L^−1^, a plot of ΔN_2_O ([N_2_O_measured_] – [N_2_O_saturation_]) vs. AOU (apparent oxygen utilization; [O_2_,_measured_] – [O_2_,_saturation_]) shows two distinct linear relationships (Fig. 4b). Linear relationship 1 (LR1) and 2 (LR2) correspond to DO concentration ranges of 240 to 22 µmol L^−1^ and 18 to 10 µmol L^−1^, respectively. The slope of LR1 (Fig. 4b) indicates that ~8500 mol of O_2_ were consumed for every mol of N_2_O produced, and this is similar to previous estimates from the open tropical Atlantic Ocean^13,14^, including the Mauritanian upwelling region^23^; it is also similar to estimates from global oxygenated oceanic water masses^8,9,29^. In contrast, the slope of LR2 implies that only 170 mol of O_2_ were consumed for every mol of N_2_O produced, and this points towards a 50-fold increase in the amount of N_2_O produced vs. the amount of DO consumed below 20 μmol O_2_ L^−1^. This result agrees with Codispoti *et al*.^34^ and Nevison *et al*.^29^ who also showed that N_2_O production starts to increase substantially below ~20 µmol O_2_ L^−1^. Our estimate of a 50-fold increase in N_2_O production vs. DO consumption is, however, higher than results from earlier work which have shown that nitrification-N_2_O yields can increase 20-fold^10^ and 40-fold^35^ under low DO concentrations. One possible explanation for our observation of higher N_2_O production vs. DO consumption could be the presence of N_2_O production via reductive pathways (i.e. sources of N_2_O production which do not also consume DO), and evidence for this is discussed below under ‘*Nitrous oxide cycling pathways*’.

Finally, simple linear regression analyses were used to quantify the N_2_O vs. DO relationships shown in Fig. 4a at DO concentrations between 250 and 20 µmol L^−1^, <20 and 10 µmol L^−1^, and <10 µmol L^−1^ (Table 1). These DO ranges were selected based on the observed N_2_O vs. DO shifts shown in Fig. 4a. The N_2_O vs. DO relationships for each of these DO ranges were then applied to the DO concentrations observed during the high resolution IFM13 glider survey in order to estimate the bulk amount of N_2_O inside the suboxic eddy core. Based on the DO concentrations measured during the glider survey (Fig. 3), the diameter of the suboxic eddy core was defined as 50 km and extended from the surface to 250 m depth, and the bulk amount of N_2_O within this volume was estimated to have been 1.8 × 10^7^ mol N_2_O, or an average of 9,200 mol N_2_O km^−2^. In comparison, areal N_2_O over the same depth range at OES was 5,000 mol N_2_O km^−2^, or almost half that within the suboxic eddy core. This again highlights that low oxygen eddies have the potential to be important but previously unrecognized sources of marine N_2_O. Quantifying the overall importance of low oxygen eddies is not trivial, however, and it would depend, for example, on factors such as the frequency of their occurrence, their size, and how long they last^16,36^. A recent analysis of a 1.1 × 10^6^ km^2^ area of the ETNA suggests that at any one time ~20% of this area is covered by suboxic eddy cores^16^. Assuming all of these suboxic eddy cores are similar to the one described here, which showed an almost 100% increase in N_2_O concentrations inside vs. outside of the eddy, this could require bulk upper water column (in this case upper 250 m) N_2_O estimates to be increased by up to 20%. This is a first order estimate, however, and much more shipboard work is necessary to accurately determine the DO and N_2_O conditions within a range of ETNA eddies, and covering their full lifecycles, so that more robust statistical analyses of their potential importance as a source of marine N_2_O can be calculated. Furthermore, if the prevalence of suboxic eddies are also found to be high outside of the ETNA, these types of low oxygen events may be found to be important at the global scale, rather than just the regional scale.

**Table 1:** Results from simple linear regression analyses of N_2_O vs. DO at different DO concentration ranges.

Dissolved O_2_ Concentration Range	N_2_O (nmol L^−1^) vs. O_2_ (µmol L^−1^) Linear Regression Equation	*p*-value	R^2^-value
250–≥20 µmol L^−1^	N_2_O_conc._ = −0.162 × O_2 conc._ + 45.48	<0.001	0.97
<20–10 µmol L^−1^	N_2_O_conc._ = −6.67 × O_2 conc._ + 179.04	0.03	0.95
10–5 µmol L^−1^	N_2_O_conc._ = 4.30 × O_2 conc._ + 71.28	—	—

The results shown here were obtained using data shown in Fig. 2a. The regression equations were used in conjunction with the DO concentrations measured during the IFM13 glider survey of the eddy (see *Methods* section) in order to estimate the bulk amount of N_2_O inside the suboxic eddy.

### Nitrous oxide cycling pathways

**Figure 5:**
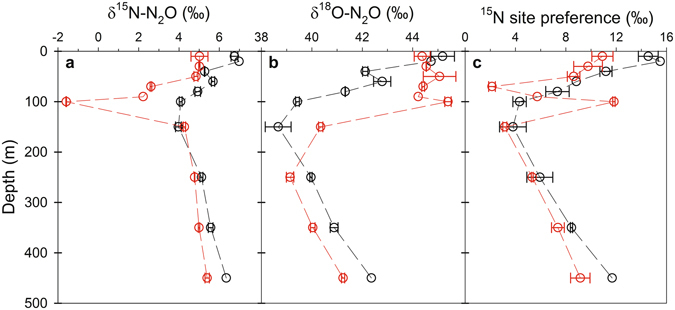
Vertical distributions of (**a**) δ^15^N-N_2_O, (**b**) δ^18^O-N_2_O and (**c**) ^15^N site preference signatures at ES1 (red circles) and OES (black circles). Error bars represent the standard deviation of duplicate N_2_O isotope measurements. Due to the loss of duplicated samples, standard deviations are not reported for 20 and 450 m depth at OES, and 90 m depth at ES1.

**Figure 6:**
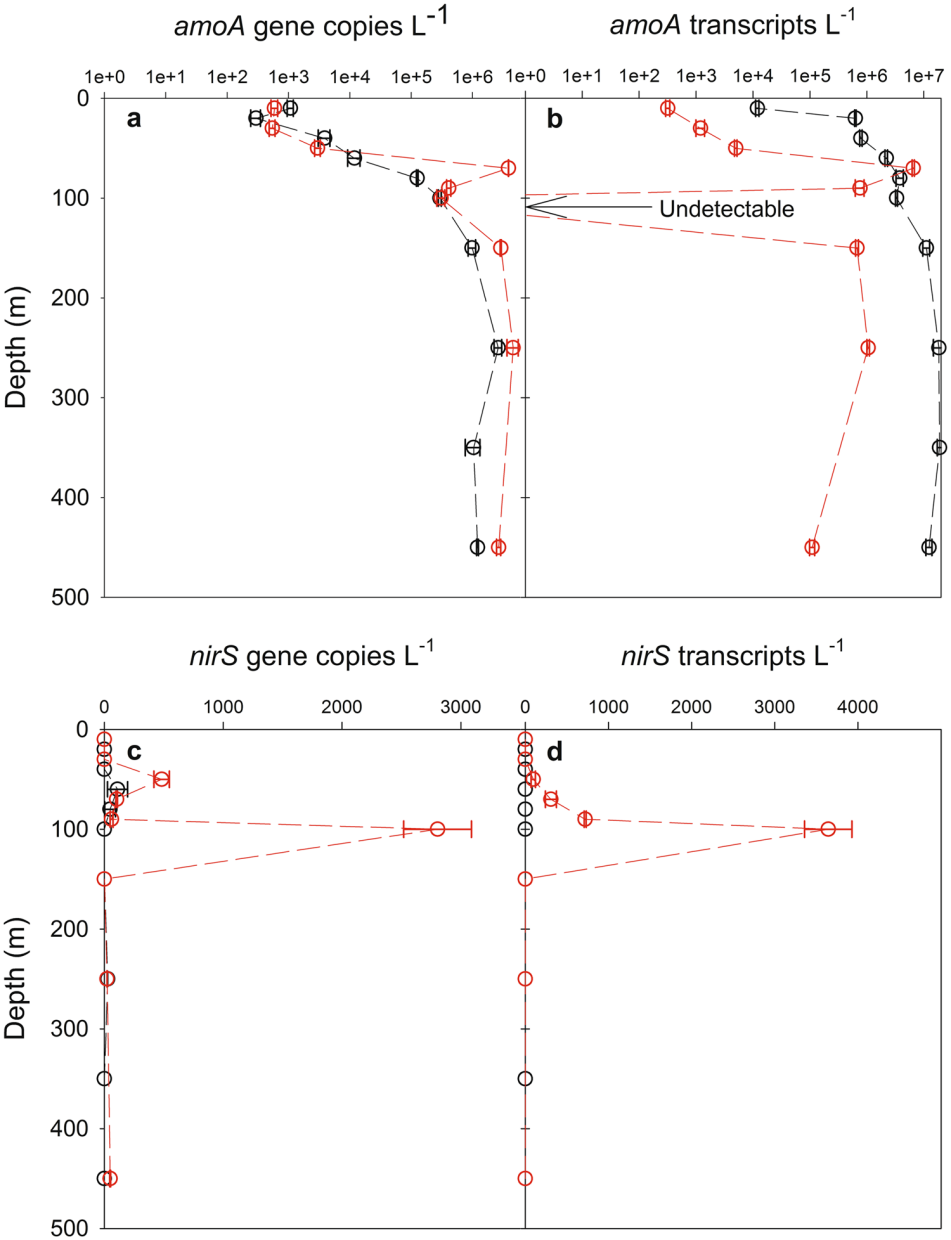
Vertical distributions of (**a**) *amoA* gene copies, (**b**) *amoA* transcripts, (**c**) *nirS* gene copies and (**d**) *nirS* transcripts at ES1 (red circles) and OES (black circles). The error bars represent the standard deviation of duplicate measurements. Note: the *amoA* and *nirS* gene data were also included in a description of the microbial community within the eddy by Löscher *et al*.^21^.

Results from isotope and isotopomer (i.e. the intramolecular distribution of ^15^N within the linear NNO molecule; δ^15^N^α^ - δ^15^N^β^) measurements, as well as molecular genetic analyses, point towards shifts in the N_2_O cycling pathways in the core of the suboxic eddy, relative to the more oxygenated waters inside and outside of the eddy. This complete suite of isotope, isotopomer (^15^N site-preference; SP) and molecular genetic sampling was not conducted at ES2, so our discussion focuses on ES1 with comparisons to OES. At OES, vertical profiles of δ^15^N^bulk^-N_2_O, δ^18^O-N_2_O and SP (Fig. 5) were characteristic of those from regions of the tropical South Atlantic, indicating that N_2_O was produced by a combination of AO and nitrifier-denitrification^24^. The δ^15^N^bulk^-N_2_O:SP ratios were also within the range of those reported for N_2_O produced via nitrification processes^28^. Furthermore, gene copy numbers and transcripts, which provide an indication of gene abundance and expression, respectively, of *amoA* and *nirS* genes can also provide insight into the potential N_2_O cycling pathways. The *amoA* gene is the classical functional marker gene encoding for a subunit of the ammonia monooxygenase enzyme which catalyzes AO, and a correlation between N_2_O formation by AO and *amoA* gene expression has been previously demonstrated^37,38^. To this end, we consider it reasonable to connect at least the potential for N_2_O formation to *amoA* abundance and expression. In contrast, the *nirS* gene encodes for the enzyme involved in NO_2_^−^ reduction via the denitrification pathway, and recent results have shown a positive relationship between the abundance of *nirS* genes and N_2_O production by denitrification^35^. At OES, *amoA* gene abundance and expression (Fig. 6a,b) were considerably higher than that of the *nirS* gene (Fig. 6c,d). This supports our assertion that nitrification was the major source of N_2_O within the oxygenated waters of OES, and it is consistent with previous studies in this region^13^. Similarly, above and below the ES1 DO minimum, nitrification processes also appear to be the predominant source of N_2_O, as δ^15^N^bulk^-N_2_O and SP ratios were again within the range expected for N_2_O produced by nitrifiers^28^, and *amoA* gene abundance and expression were high. The SP signatures above and below the ES1 DO minimum were, however, in some cases somewhat lower than those found at OES (Fig. 5c). Both nitrifier-denitrification and denitrification yield N_2_O with an SP ≤0‰, whereas AO yields N_2_O with an SP >30‰^4,39,40^. The SP observations therefore indicate that a reductive pathway (i.e. nitrifier-denitrification or denitrification), rather than an oxidative pathway, was a relatively more important source of N_2_O at ES1 vs. OES. Given that *amoA* gene abundance was high above and below the ES1 DO minimum, whereas *nirS* gene abundance and expression were either undetectable or very low, we suggest that nitrifier-denitrification was the most probable reductive N_2_O production pathway above and below the ES1 DO minimum. The potential increase in N_2_O production via nitrifier-denitrification at ES1, when compared to OES, was likely a result of the lower DO concentrations inside vs. outside of the suboxic eddy, as culture investigations have shown that nitrifier-denitrification increases as DO concentrations decrease^4^.

Differences in isotope and molecular genetic results inside vs. outside of the suboxic eddy were most prominent within the ES1 DO minimum at 100 m, and our results provide insights into how N_2_O cycling changed as the DO concentration dropped to ~10 μmol L^−1^. One of the most prominent differences was the δ^15^N^bulk^-N_2_O value, which decreased to −1.6‰ within the ES1 OMZ. This is below the lowest value reported for the Arabian Sea^31^, and is one of the lowest values reported for seawater, one exception being the Black Sea where a value of −10.8‰ was observed^41^. Westley *et al*.^41^ concluded that this ^15^N^bulk^-N_2_O value of −10.8‰ was too low to have been caused by a reductive N_2_O production pathway, and instead they concluded that it must have been caused by N_2_O produced via AO. Although very low for seawater, the δ^15^N^bulk^-N_2_O value we observed within the ES1 DO minimum was not as extreme as that of Westley *et al*.^41^, and taken alone it cannot be used to narrow down the predominant production pathway. That is, based on the δ^15^N signatures of dissolved inorganic nitrogen from the eastern tropical Atlantic^13^, and the range of measured isotope effects of nitrification and denitrification (summarized by Bange)^2^, the δ^15^N^bulk^-N_2_O value from the ES1 DO minimum could have been produced by either oxidative or reductive pathways. Our molecular genetic results, however, provide further insight into the potential predominant N_2_O production pathway. The abundance and expression of *nirS* genes increased substantially within the ES1 OMZ (Fig. 6c,d), and these results, particularly the increase in *nirS* gene expression, suggest that denitrification was actively occurring at the ES1 DO minimum. In contrast, although *amoA* gene abundance was still relatively high (Fig. 6a), the expression of *amoA* genes became undetectable within the ES1 DO minimum (Fig. 6b), thus indicating a substantial reduction in N_2_O production via AO. Ultimately, our molecular genetic results provide evidence that implies that denitrification became an important source of N_2_O within the ES1 DO minimum. Our suggestion that denitrification was an important source of N_2_O at DO concentrations ~10 μmolL^−1^ (i.e. ES1 OMZ) is also supported by a recent modelling study^25^ and by ^15^N tracer measurements^35^ which found that denitrification was an important source of N_2_O at similar DO concentrations.

While it appears that denitrification was an important source of N_2_O within the ES1 OMZ, two lines of evidence point towards the potential for at least partial N_2_O consumption by denitrification. Firstly, in comparison to the more oxygenated waters directly above and below it, the δ^15^N^bulk^-N_2_O and δ^18^O-N_2_O values at the ES1 DO minimum decreased and increased, respectively (Fig. 5a,b). The observation of a concomitant δ^15^N^bulk^-N_2_O decrease and δ^18^O-N_2_O increase is extremely rare, however, it has been observed in the Black Sea where it was interpreted as indicating simultaneous N_2_O production and consumption (i.e. a production source which decreases δ^15^N^bulk^-N_2_O and a consumption sink which increases δ^18^O-N_2_O)^41^. Secondly, a notable SP increase was also observed in the ES1 DO minimum (Fig. 5c). Similar to nitrifier-denitrification, production of N_2_O by denitrification yields N_2_O with an SP ≤0‰, while AO produces N_2_O with an SP ≥30‰^4,39,40^. As such, an initial interpretation of the SP result by itself could point towards a larger contribution of N_2_O via the AO route. Given the extremely reduced expression of *amoA* genes in the ES1 DO minimum, however, this seems unlikely. Instead, reduction of N_2_O to N_2_ can also result in an SP increase, albeit a highly variable one^42–44^, and results have shown that the reduction of N_2_O can cause SP to increase even when there are net N_2_O gains^45^. Based on the simultaneous δ^15^N^bulk^-N_2_O decrease and δ^18^O-N_2_O increase, and the increased SP signature, we therefore propose that some of the N_2_O produced within the ES1 DO minimum was subsequently reduced to N_2_.

Although limited in scope, this is the first study to show evidence which points towards N_2_O cycling by denitrification in the open Atlantic Ocean. As outlined earlier, ~20% of the ETNA is covered by low oxygen eddy cores at any one time; as such, if denitrification is also cycling N_2_O in these other low oxygen eddies, we may need to change our classical view that N_2_O cycling in the ETNA is restricted to nitrification. It is, however, important to point out that while we have suggested that denitrification played a role in cycling N_2_O within the suboxic eddy investigated during this study, it only appears to be important at the nanomolar scale (i.e. the scale at which we measure N_2_O), as at the micromolar scale there was no evidence for NO_3_^−^ reduction^20^ or biogenic N_2_ production (Altabet and Grundle, unpublished data) inside the suboxic eddy. Still, some of the low oxygen eddies which have been observed in the ETNA with moored and glider based instruments have been characterized by DO concentrations even lower than those reported here^15^, and to this end, it is possible that some of the low oxygen eddies in the ETNA may also be sites of fixed N losses at the micromolar scale.

## Summary

The present study has demonstrated for the first time that low DO eddies in the eastern tropical North Atlantic can cause significant shifts in the N_2_O cycling dynamics which are typically found in this region. Furthermore, this work has shown that low DO eddies can serve as ideal ‘natural laboratories’ for investigating the impact of decreasing DO concentrations for marine N_2_O conditions. In the case of this study, our results showed that at DO concentrations <~20 µmol L^−1^, N_2_O production increased substantially, resulting in concentrations which were within the range of many of the highest N_2_O concentrations reported for major OMZ regions such as the open Arabian Sea and eastern tropical Pacific. This result has demonstrated the magnitude by which N_2_O production could increase if open ocean DO concentrations decrease in the tropical Atlantic. Isotope and molecular genetic results also provided evidence for a major shift in the N_2_O cycling pathways at ~10 μmol O_2_ L^−1^, such that it appeared that denitrifcation not only started to produce N_2_O, it also started to partially consume some of the N_2_O. Finally, as DO concentrations decreased to ~5 μmol L^−1^ the N_2_O concentration also decreased, possibly indicating a switch from net N_2_O production to net N_2_O consumption. Ultimately, given that this study has shown the capacity of low DO eddies to be N_2_O production ‘hotspots’, and because N_2_O cycling pathways (i.e. denitrification) not previously thought to occur in the Atlantic were observed, a reevaluation of N_2_O budgets and cycling in the tropical Atlantic Ocean may be necessary.

## Methods

### Glider Surveys

Two Slocum gliders (IFM12 and IFM13) manufactured by Teledyne Webb Research were used in this study to observe the temperature, salinity, dissolved oxygen and current fields north of the Cape Verde archipelago. Between January and April 2014, these gliders were able to confirm the presence of an anti-cylconic mode-water eddy, which had formed off the coast of Mauritania and then propagated westward toward the Cape Verde Ocean Observatory (CVOO; Fig. 1). IFM12 was deployed on January 10^th^, 2014 from the Cape Verdean *RV Islandia*, and it first entered the eddy reported here on January 23^rd^, 2014. A first section through the eddy core was completed by IFM12 on February 3^rd^ 2014, and results confirmed that it was a low DO eddy. IFM13 was deployed from *RV Meteor* on March 17^th^, 2014 and completed a section through the eddy core on April 7^th^, 2014. The data collected by the gliders underwent post-processing routines that included a glider-speed dependent thermal lag correction of the conductivity cell^46^, and a mixed lab/*in-situ* calibration of the Aanderaa Optode oxygen sensor. Finally, the locations of the eddy during the IFM12 and IFM13 deployments, and the locations of the glider sections are shown in Fig. 1, and DO concentrations from the IFM13 section through the eddy are shown in Fig. 3.

### Ship-Based Sampling

Between March 6^th^ and 7^th^ 2014, the *RV Islandia* was used to conduct sampling for a suite of biological, chemical and physical parameters at a station inside the suboxic eddy (eddy station 1; Fig. 1). In order to allow for comparisons between measurements made inside the eddy with conditions outside the eddy, the same suite of samples collected at eddy station 1 (ES1) were also collected at an outside eddy station (OES) during an *RV Islandia* cruise on February 14^th^ 2014 (Fig. 1). Here we outline the sampling and measurements of parameters that relate to N_2_O cycling.

Dissolved oxygen (DO) concentrations were measured using a Seabird SBE43 DO sensor that was attached to our conductivity, temperature and depth (CTD) profiler. The DO sensor was calibrated using DO measurements by Winkler titration on duplicated samples collected across the entire range of DO concentrations observed. The detection limit of these measurements was 3 μmol O_2_ L^−1^, and the average standard deviation of the duplicate measurements was ±0.28 μmol O_2_ L^−1^. It is important to note, however, that in order to preserve Niskin bottle water for our N_2_O concentration and isotope samples, bottle samples for DO measurements by Winkler titration were not collected on our N_2_O vertical sampling casts. Samples for DO measurements by Winkler titration, for the purpose of calibrating the DO sensor, were instead collected on CTD casts immediately before and after our N_2_O sampling casts. The average standard deviation between our discrete Winkler DO measurements and our calibrated CTD-DO sensor measurements was ±1.31 μmol O_2_ L^−1^. Considering the errors involved in both our duplicated Winkler DO measurements (±0.28 μmol O_2_ L^−1^) and our CTD-DO measurements (±1.31 μmol O_2_ L^−1^), the average propagation of error associated with the DO concentrations we report for eddy station 1 is ±1.33 μmol O_2_ L^−1^. Discrete depth water samples were also collected from the surface to 450 m depth for the purpose of measuring N_2_O concentrations, isotope and isotopomer signatures of N_2_O, and for quantifying the abundance and transcripts of ammonia-monooxygenase genes of nitrifying bacteria and archaea, and nitrite reductase genes of denitrifying bacteria (*amoA* and *nirS*, respectively; all protocols described below).

On March 18^th^ 2014, we also conducted a CTD-DO survey and collected water samples for N_2_O concentration measurements at an additional inside eddy station (eddy station 2; Fig. 1) on the *RV Meteor* cruise M105. The CTD-DO sensor was calibrated following the same protocols outlined above, and the standard deviation of duplicate DO measurements by Winkler titration was ±0.35 μmol L^−1^, while the average standard deviation between our discrete Winkler DO measurements and our CTD-DO sensor measurements was ±1.23 μmol L^−1^. To this end, the average propagation of error associated with the DO concentrations we report for eddy station 2 is ±1.28 μmol L^−1^.

### N_2_O concentration, and isotope/isotopomer measurements

Water samples for N_2_O concentration, and isotope and isotopomer measurements were collected in duplicate 60 ml and 120 ml serum bottles, respectively, following standard dissolved gas sampling protocols^47^. Immediately following collection, the samples were poisoned with 100 µl of a saturated HgCl_2_ solution and then stored until analysis ashore.

N_2_O concentration samples were stored for ~2 months prior to being measured on a gas chromatograph with an attached electron capture detector using the headspace equilibration method described by Grundle *et al*.^47^. Final dissolved N_2_O concentrations were calculated using corresponding measurements of *in situ* temperature and salinity, corrected for temperature and pressure during the headspace equilibration following the solubility tables of Weiss and Price^48^. The average standard deviation of our duplicate N_2_O concentration measurements was ±0.8 nmol L^−1^.

Isotope (δ^15^N^bulk^-N_2_O vs. AIR and δ^18^O-N_2_O vs. VSMOW) and isotopomer (δ^15^N^α^-N_2_O and δ^15^N^β^-N_2_O vs. AIR) analysis began with continuous helium (He) gas stripping of dissolved N_2_O out of samples as described in Charoenpong *et al*.^49^. Briefly, sample water was pumped in and out of a gas extractor (14 ml min^−1^) through which He was constantly bubbled (90 ml min^−1^). Quantitative yield was verified by comparison of N_2_O recovery from seawater with known N_2_O concentration (established by atmospheric equilibration) and with standard gas injected directly into the He gas flow. Following extraction, the method of McIlvin and Casciotti^50^ was followed in which a purge/trap system was used to purify and concentrate extracted N_2_O. This included two-step cryofocusing with passage through CO_2_ and H_2_O traps as well as a 30 m × 0.53 mm GS-Q capillary column. Sample N_2_O was introduced via continuous He carrier flow into a multicollector IsoPrime isotope ratio mass spectrometer (IRMS). Masses 44, 45, and 46, and masses 30 and 31 which arise from the NO+ fragment of N_2_O which is formed in the ion source, were monitored, and sample N_2_O was detected as a well-resolved sharp peak which was bracketed by broader reference N_2_O peaks. The 45/44 and 46/44 peak areas were used to derive δ^18^O-N_2_O and δ^15^N^bulk^-N_2_O, respectively, while the 31/30 peak area was used to derive δ^15^N^α^-N_2_O. The δ^15^N^bulk^-N_2_O and δ^15^N^α^-N_2_O values were used to calculate δ^15^N^β^-N_2_O. Calibration of δ^15^N^α^-N_2_O, δ^15^N^β^-N_2_O and δ^18^O-N_2_O was accomplished using 4 certified standard gases (supplied by Joachim Mohn)^51^ that ranged widely in these values and encompassed those reported here. Calibration for N_2_O site-specific isotopomer composition also needs to account for instrument specific ‘scrambling’ in the mass spectrometer ion source between ^15^N^14^NO and ^14^N^15^NO^50^. The magnitude is on the order of 10% and is manifested as changes in the 30/44 ratio from the value expected in the absence of scrambling. In order to account for this, we took advantage of new standard materials that vary widely in isotopomer composition^51^ to perform an empirical curve-fitting calibration. Finally, based on measurements of duplicate samples from each sampling depth, the errors associated with our isotope measurements were ±0.07, 0.17, 0.36 and 0.18‰ for δ^15^N^bulk^-N_2_O, δ^15^N^α^-N_2_O, δ^15^N^β^-N_2_O and δ^18^O-N_2_O, respectively.

### Molecular genetic analyses

Water samples (~2 L) were filtered through 0.2 µm polyethersulfone membrane filters, which were immediately stored at −80 °C until nucleic acid purification was performed ashore following Löscher *et al*.^38^. RNA was treated with Dnase to remove any residual DNA, and RNA purity was verified by non-template quantitative-PCRs for *amoA* (ammonia monooxygenase) and *nirS* (nitrite reductase) genes. Reverse transcription was performed following Löscher *et al*.^38^. Quantitative-PCRs of bacterial and archaeal *amoA* were performed in technical duplicates with standards obtained from *Nitrosococcus oceani NC1* and from an environmental clone for archaeal *amoA*^38^, while the same was achieved for *nirS* using a standard obtained from Paracoccus denitrificans (Pd 1222)^38^. All reactions were performed in a volume of 12.5 µl using a ViiA7 quantitative-PCR system following the protocols and PCR conditions outlined by Löscher *et al*.^38^ and Lam *et* *al*.^52^.
