# Author Correction: Low oxygen eddies in the eastern tropical North Atlantic: Implications for N_2_O cycling

**DOI:** 10.1038/s41598-018-22120-3

**Published:** 2018-03-01

**Authors:** D. S. Grundle, C. R. Löscher, G. Krahmann, M. A. Altabet, H. W. Bange, J. Karstensen, A. Körtzinger, B. Fiedler

**Affiliations:** 1 GEOMAR Helmholtz Centre for Ocean Research Kiel, Kiel, Germany; 2 Bermuda Institute of Ocean Sciences, Saint George’s, Bermuda; 3 University of Southern Denmark, Odense, Denmark; 4 School for Marine Science and Technology, University of Massachusetts Dartmouth, Dartmouth, USA

Correction to: *Scientific Reports* 10.1038/s41598-017-04745-y, published online 06 July 2017

This Article contains errors in Reference 36 which was incorrectly given as:

Schütte, F., Brandt, P. & Karstensen, J. Occurrence and characteristics of mesoscale eddies in the tropical northeast Atlantic Ocean. *Ocean Sci. Discuss* **12**, 3043–3097 (2015).

The correct reference is listed below as ref. 1:
